# Protection of Pyruvate against Glutamate Excitotoxicity Is Mediated by Regulating DAPK1 Protein Complex

**DOI:** 10.1371/journal.pone.0095777

**Published:** 2014-04-22

**Authors:** Jingwei Tian, Jucan Cheng, Jianzhao Zhang, Liang Ye, Fangxi Zhang, Qiuju Dong, Hongbo Wang, Fenghua Fu

**Affiliations:** Key Laboratory of Molecular Pharmacology and Drug Evaluation (Ministry of Education of China), School of Pharmacy, Yantai University, Yantai, China; University of Iowa, United States of America

## Abstract

The neuroprotective activity of pyruvate has been confirmed in previous in vivo and in vitro studies. Here, we report a novel mechanism that pyruvate prevents SH-SY5Y cells from glutamate excitotoxicity by regulating death-associated protein kinase 1 (DAPK1) protein complex. Our results showed pyruvate regulated DAPK1 protein complex to protect cells by two ways. First, pyruvate induced the dissociation of DAPK1 with NMDA receptors. The disruption of the DAPK1-NMDA receptors complex resulted in a decrease in NMDA receptors phosphorylation. Then the glutamate-stimulated Ca^2+^ influx was inhibited and intracellular Ca^2+^ overload was alleviated, which blocked the release of cytochrome c and cell death. In addition, increased Bcl-xL induced by pyruvate regulated Bax/Bak dependent death by inhibiting the release of cytochrome c from the mitochondrial inter-membrane space into the cytosol. As a result, the cytochrome c-initiated caspase cascade, including caspase-3 and caspase-9, was inhibited. Second, pyruvate promoted the association between DAPK1 and Beclin-1, which resulted in autophagy activation. The autophagy inhibitor 3-methyladenine reversed the protection afforded by pyruvate. Furthermore, the attenuation of mitochondrial damage induced by pyruvate was partly reduced by 3-methyladenine. This suggested autophagy mediated pyruvate protection by preventing mitochondrial damage. Taken together, pyruvate protects cells from glutamate excitotoxicity by regulating DAPK1 complexes, both through dissociation of DAPK1 from NMDA receptors and association of DAPK1 with Beclin-1. They go forward to protect cells by attenuating Ca^2+^ overload and activating autophagy. Finally, a convergence of the two ways protects mitochondria from glutamate excitotoxicity, which leads to cell survival.

## Introduction

Excitotoxicity, first proposed by Olney *et al.* in 1969 [1], is a pathological process evoked by excessive or prolonged activation of excitatory amino acid receptors responsible for various neurological disorders including ischemia [2], Alzheimer’s disease [3], Parkinson’s disease [4] and other neurodegenerative diseases [5]. The major excitatory neurotransmitter in the brain is glutamate, which if released too much can be destructive and excite a neuron to death in the mammalian central nervous system. More specifically, glutamate may over-activate the Ca^2+^-favoring glutamate-gated ion channels N-methyl-D-aspartate (NMDA) receptors resulting in cellular calcium overload, which consequently stimulates mitochondrial membrane depolarization [6], caspase activation [7], nitrogen free radicals production [8], and eventually leads to cell death [9], [10]. Recently, more attentions have been given to the association between autophagy and excitotoxicity. Excessive autophagy may promote cell death through release of lysosomal enzymes and other factors [11]. However, autophagy on other hand is proved to be a ubiquitous cytoprotective process by selectively removing damaged mitochondria [12]. Death-associated protein kinase 1 (DAPK1) is considered to play a central role in modulating excitotoxicity and autophagy. DAPK1 can interact with NMDA receptors and stimulate their phosphorylation to mediate neuronal damage [13]. Meanwhile, it binds to Beclin-1, resulting in its dissociation from Bcl-2 family [14] and allowing Beclin-1to induce autophagy [15]. With the evidence that DAPK1-mediates injury in cerebral ischemia and the ability of bioavailable DAPK1 inhibitors to rescue neuronal death, DAPK1 has emerged as an important drug-discovery target for brain disorders [16]. Efficiently regulating the interaction of DAPK1 with its partners could be applied to ameliorating brain injury.

The neuroprotective effects of pyruvate have been confirmed in many neurological disorders like ischemia [17], Alzheimer’s disease [18] and Parkinson’s disease [19]. The neuroprotective mechanisms of pyruvate are known to be through antioxidation [20], [21], anti-inflammation [22] and induction of endogenous erythropoietin (EPO) expression [17]. However, the protective effect of pyruvate by regulating DAPK1 complex has not been investigated. Therefore, this current study is aimed to explore a novel cytoprotective mechanism of pyruvate on excitotoxicity mediated by regulating DAPK1 and its interacting proteins.

## Materials and Methods

### Materials

Pyruvate was obtained from Sinopharm Chemical Reagent Co.Ltd (Shanghai, China) with a purity of more than 98%. 3-Methyladenine was purchased from Sigma-Aldrich (St. Louis, MO, USA). Anti-Bcl-xL antibody was obtained from Santa Cruz Biotechnology (CA, USA). The antibodies against Beclin-1, DAPK1, NMDA receptor, p-NMDA receptor, Bcl-2, Bax, caspase-3, caspase-9, cytochrome c and β-actin were purchased from Cell Signaling Technology (Boston, MA, USA). Polyclonal antibody of NMDA receptor was produced by immunizing animals with a synthetic phosphopeptide corresponding to residues surrounding serine 890 of human NMDAR1 [23]. JC-1 mitochondrial membrane potential assay kit, cell buffer for western blotting and immunoprecipitation and fluo-3/AM were purchased from Beyotime Institute of Biotechnology (Shanghai, China). SH-SY5Y cells were purchased from the Type Culture Collection of the Chinese Academy of Sciences (Shanghai, China). (4Z)-4-(3-Pyridylmethylene)-2-styryl-oxazol-5-one was purchased from (Merck KGaA, Darmstadt, Germany). Other reagents were obtained from Sigma-Aldrich Co. (St. Louis, MO, USA) unless indicated otherwise.

### Cell Culture

SH-SY5Y cells were widely selected as a glutamate excitotoxicity model system because of well represent a model system for some aspects of neurotoxicity [24]. SH-SY5Y cells were maintained in complete DMEM media (containing 10% FBS, 100 U/ml penicillin and 100 µg/ml streptomycin) at 37°C in a humidified atmosphere of 5% CO_2_ in air.

### Cell Viability Assay

The viabilities of SH-SY5Y cells were determined by MTT assay [25]. SH-SY5Y cells were seeded into 96 well plates at a density of 4×10^4^ cells/well. After treatment, the cells were incubated with 5 mg/ml MTT for 4 hours. Then the medium was replaced with DMSO and absorbance values were determined at 570 nm. The cell viability (%) was calculated using the formula: [A_(sample)_ – A_(blank)_]/[A_(control)_ – A_(blank)_]×100%, %, where A_(sample)_, A_(control)_ and A_(blank)_ are the absorbance of cells.

### Flow Cytometric Measurement of [Ca^2+^]_i_

Intracellular calcium ([Ca^2+^]_i_) was detected by flow cytometry using the Ca^2+^ sensitive indicator fluo-3/AM [26]. Briefly, cells were challenged with glutamate for 3 hours after preincubation with pyruvate or vehicle for 30 minutes. Then the cells were washed and loaded with 3 µmol/L fluo-3/AM diluted in Krebs-Ringer buffer [10 mM D-glucose, 120 mM NaCl, 4.5 mM KCl, 0.7 mM Na_2_HPO_4_, 1.5 mM NaH_2_PO_4_, and 0.5 mM MgCl_2_ (pH 7.4 ) for 30 minutes at 37°C. Finally, cells were washed in 5 ml of Ca^2+^-free PBS at 37°C and analyzed immediately by flow cytometry after re-suspending in 1 mL of Ca^2+^ free PBS.

### Preparation of Protein and Western Blot Analysis

At the end of drug treatment, the cellular protein was prepared [27]. In brief, SH-SY5Y cells were incubated in ice-cold RIPA buffer containing protein inhibitor cocktail for 30 min. The cell suspension was centrifuged at 10,000 g for 10 min at 4°C. The supernatant was used as cell lysate and stored at −80°C before use. Protein concentrations were determined using BCA assay kit.

Protein samples were denatured with 5x sample loading buffer and separated by 10% SDS-PAGE. Then proteins were transferred on PVDF membranes. The membranes were blocked with blocking buffer (5% fat free milk powder in TBST buffer) and probed with antibodies overnight at 4°C. After incubation with horseradish peroxidase-conjugated secondary antibody, the enhanced chemiluminescence Western blotting detection reagent was added. Then the membranes were exposed to X-ray film and developed.

### Immunoprecipitation

Immunoprecipitation was performed as previous study [27]. After treatment, the cells were harvested and lysised in the cell buffer for western blotting and immunoprecipitation on ice. The lysate was then centrifuged at 12,000 g for 10 min at 4°C and the protein content of the supernatant was determined using the BCA assay. The supernatant (0.5 mg/mL) was added in 1 µg/mL IgG and 20 µl/mL protein A-Agarose. After centrifugation 1,000 g for 5 min at 4°C, the supernatant was incubated with 10 µl/ml anti-DAPK1 antibody at 4°C for 1 hour. Then 20 µl/ml protein A-Agarose was added to the lysate and mixed at 4°C overnight. After centrifugation 1,000 g for 5 min at 4°C, the supernatant was removed. The pellet was washed 4 times and a portion taken for protein concentration determination. The remaining pellet was re-suspended with loading buffer. The sample was boiled for 5 min, and then centrifuged at 1,000 g for 5 min at 4°C. Finally, obtained samples were analyzed by western blot analysis with anti-NMDA receptor or Beclin-1 antibody.

### Mitochondrial Membrane Potential Assay

Mitochondrial membrane potential was detected by the JC-1 mitochondrial membrane potential assay kit [28]. In brief, cells were seeded in 6-well plate. After treatment, cells were washed with PBS. Then JC-1 working solution and culture medium were added. After 20 minutes incubation at 37°C, cells were washed with JC-1 staining buffer. Then culture medium was added and the fluorescence was measured by fluorescence microscopy.

### Statistical Analysis

All results were expressed as means ± SEM and repeated at least 3 times independently. Inter-group difference of means of quantitative parameters was statistically analyzed by using one way analysis of variance (ANOVA).

## Results

### Pyruvate Attenuated Glutamate-induced Cytotoxicity

**Figure 1:**
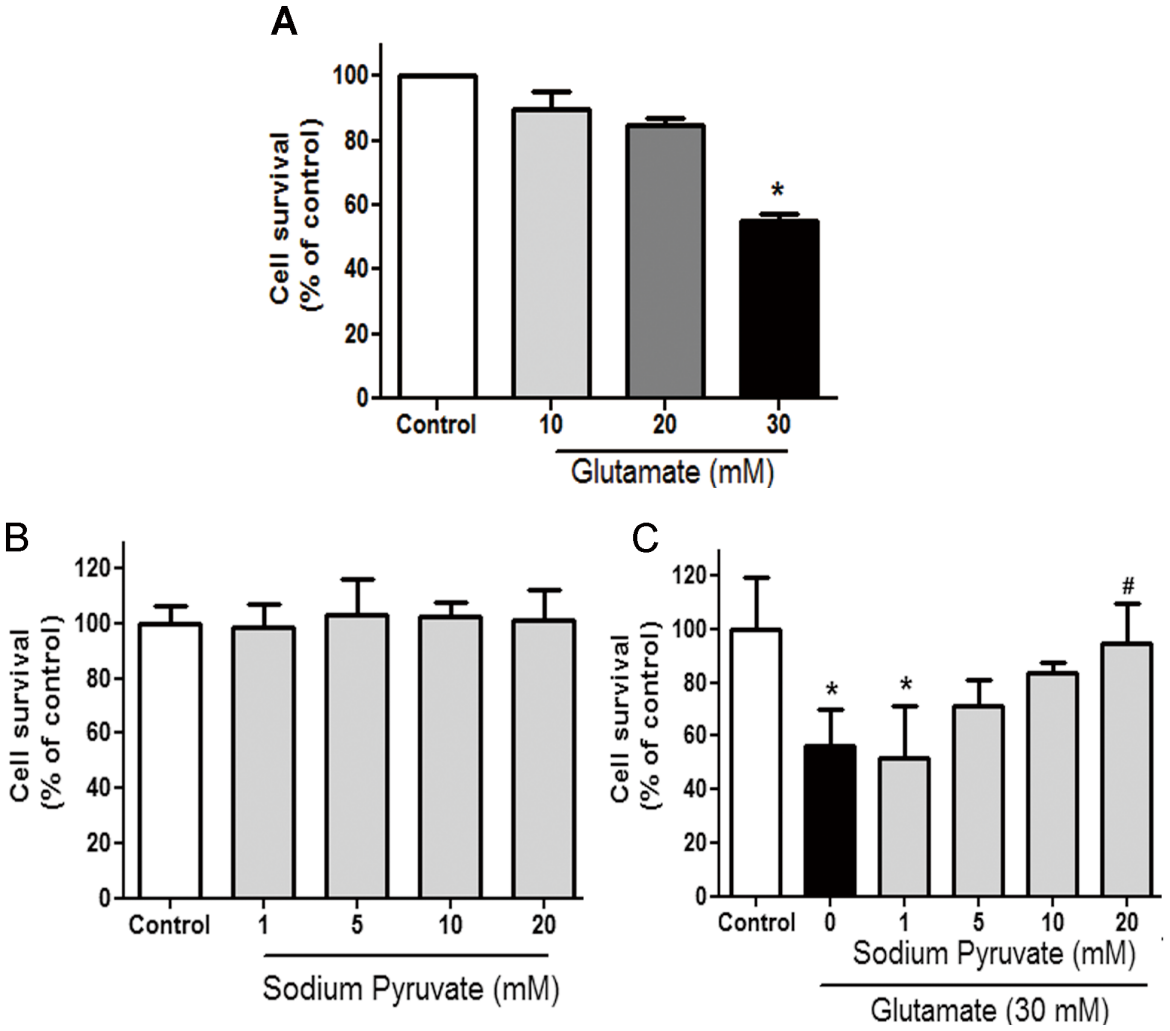
Pyruvate protected cells against glutamate insult. Cell survival was determined by MTT assay. A, Cells were treated with 10, 20 or 30°C. Cell survival of control cells was set as 100%. *P<0.05 vs control; ^#^ *P*<0.05 vs glutamate, n = 6.

The viabilities of SH-SY5Y cells with or without pyruvate protection were determined by MTT assay. The cells presented a concentration-dependent reduction in their survival rates when incubated with different concentrations of glutamate (10, 20 and 30 mM) for 3 hours, especially 56.1% of survival in 30 mM glutamate group (Fig. 1A). Pyruvate alone (5–20 mM) did not affect the growth of SH-SY5Y cells after 30 minutes exposures (Fig. 1B). However, a concentration-dependent increase in cell viability was noted when cells were pre-incubated with pyruvate at different concentrations followed by glutamate incubation. Cell viability was up to 94.5% in 20 mM pyruvate treatment group, which was significantly higher than that of exposure to glutamate alone (Fig. 1C). These results indicated that glutamate induced cytotoxicity could be remarkably attenuated by pyruvate pre-treatment.

### Pyruvate Stimulated DAPK1 Dissociation from NMDA Receptor but Association with Beclin-1

**Figure 2:**
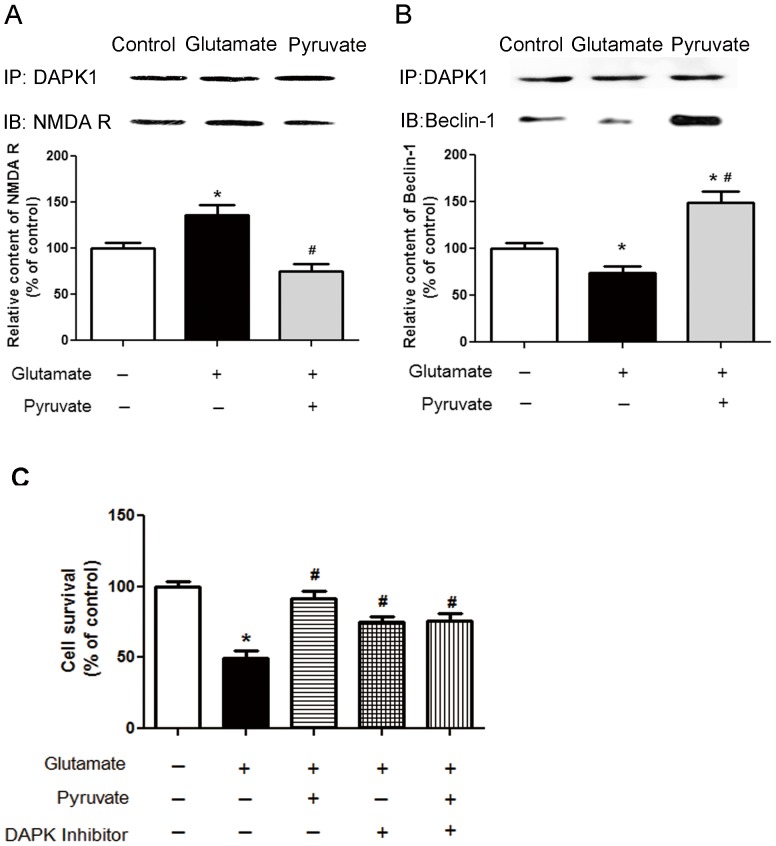
Pyruvate induced DAPK1 dissociation from NMDA receptor and association with Beclin-1. Cells were pretreated with vehicle or 20°C. After preincubation, cells were challenged with vehicle or 30 mM glutamate for 3 hours. After treatment cells were lysed and the lysate was immunoprecipitated by anti-DAPK1 antibody. Then the immunoprecipitated proteins were detected with anti-NMDA receptor antibody (A) or anti-Beclin-1 antibody (B). All the immunoprecipitated protein was determined by anti-DAPK1 antibody (IP). The protein levels that were pulled down by DAPK1 in control cells were set as 100%. (C), Cell survival was determined by MTT assay. Cells were treated with vehicle, pyruvate for 30 minutes or preincubation with 100 nM DAPK1 inhibitor for 1 hour followed by 30 minutes pyruvate exposure. Then cells were challenged with 30 mM glutamate for 3 hours. All cells were cultured at 37°C. Cell survival of control cells was set as 100%. *P<0.05 vs control; ^#^ *P*<0.05 vs glutamate; n = 3.

DAPK1 interaction with NMDA receptor was determined in SH-SY5Y cells (Fig. 2). When SH-SY5Y cells were challenged with glutamate, the amount of DAPK1-combined NMDA receptors were significantly increased to 136.1% compared with the control, which was reduced when pre-incubation with pyruvate (Fig. 2A). Meanwhile, DAPK1’s binding to Beclin-1 in SY5Y cells was reduced to 74.0% by glutamate exposure, which was reversed to 148.9% after pyruvate pre-treatment (Fig. 2B). These results demonstrated that pyruvate prevented the formation of DAPK1- NMDA receptor complex but stimulated the interaction between DAPK1 and Beclin-1. To verify the role of DAPK kinase in glutamate-induced excitotoxicity, a selective DAPK1 inhibitor, (4Z)-4-(3-Pyridylmethylene)-2-styryl-oxazol-5-one was adopted. Cell survival rate was increased from 49.5% to 75.3% when the cells were pre-incubated with 0.1 µM DAPK1 inhibitor for 1 hour followed by a 3-hour glutamate exposure. The protective effect of DAPK1 inhibitor was lower than that of pyruvate. The combined treatment with DAPK1 inhibitor and pyruvate caused cell viability to increase to 75.7%, which was approximate to DAPK1 inhibitor monotherapy group (Fig. 2C). In the presence of DAPK1 inhibitor, the protection of pyruvate was partly blocked. These results indicated that pyruvate protected cells from glutamate excitotoxicity mediated by DAPK1.

### Pyruvate Attenuated NMDA Receptors Phosphorylation and Calcium Overload Induced by Glutamate

**Figure 3:**
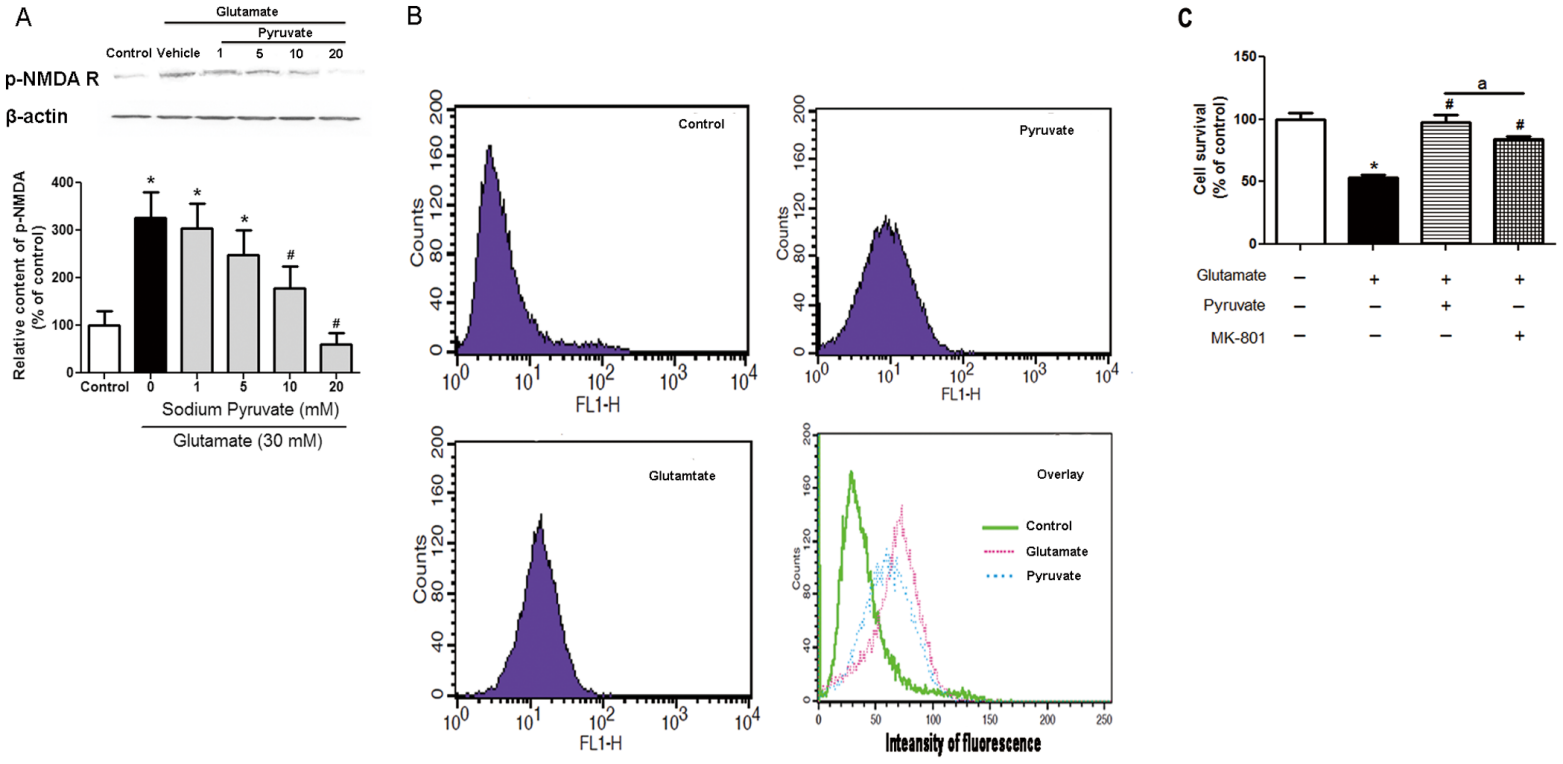
The protection of pyruvate was mediated by NMDA receptors. A, Cells were pretreated with vehicle or pyruvate (from 1 to 20 mM) for 30 minutes at 37°C. After preincubation, cells were challenged with vehicle or 30 mM glutamate for 3 hours. The phosphorylation of NMDA receptor was determined by western blot analysis. β-Actin was used as an internal control. The p-NMDA receptor/Actin ratio in control cells was set as 100%. B, Intracellular free calcium concentration was measured by flow cytometry using the Ca^2+^ sensitive indicator fluo-3/AM. Total 2×10^4^ cells were analyzed for each sample. Cells in control group were only treated with vehicle. Cells in pyruvate group were preincubated with pyruvate and then were challenged with glutamate. Cells in model group were challenged with glutamate. C, Cell survival was determined by MTT assay. Cells were treated with vehicle, 20 mM pyruvate or 1 µM MK-801 followed by glutamate exposure for 3 hours. Cell survival of control cells was set as 100%. *P<0.05 vs control; ^#^ *P*<0.05 vs glutamate; a *P*<0.05 vs pyruvate; n = 3.

NMDA receptor can be phosphorylated and activated by DAPK1 [13]. The increased interaction between NMDA receptor and DAPK1 induced by glutamate suggested the phosphorylation of NMDA receptor could be enhanced. Therefore, NMDA receptors phosphorylation was determined by western blot. About 3.3-fold NMDA receptors phosphorylation was examined after glutamate exposure, which could be concentration-dependently weakened after pre-treatment with 1 to 20 mM pyruvate (Fig. 3A). Meanwhile, exposure to glutamate triggered a significant elevation in [Ca^2+^]_i_, which was remarkably attenuated when the cells were preincubated with pyruvate (Fig. 3B). The potent inhibitor of NMDA subtype of glutamate receptors MK-801 was used in the experiments. The highest cell survival rate was 77.7% after pre-treatment with 1 µM MK-801. Interestingly, pyruvate displayed stronger protective effects than MK-801 (Fig. 3C). This suggested that the protective effect of pyruvate could be attributed to reduced NMDA receptors phosphorylation and consequently lower calcium overload. However, the involvement of other signaling pathways could not be excluded.

### Pyruvate Protected Cells through Bcl-xL/Bax/cytochrome c/caspase-3 Pathway

**Figure 4:**
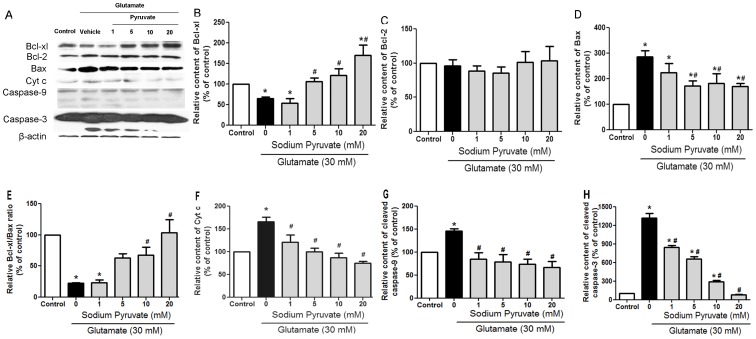
Pyruvate activated Bcl-xL-cytochrome c-caspase9/3 pathway. Cells were pretreated with vehicle or pyruvate (from 1 to 20 mM) for 30 minutes at 37°C. After preincubation, cells were challenged with vehicle or 30 mM glutamate for 3 hours. Bcl-xL, Bcl-2, Bax, cytochrome c, caspase-9 and caspase-3 levels were determined by western blot analysis. β-actin was used as an internal control. B, C, D, E, F, G and H represented Bcl-xL, Bcl-2, Bax, Bcl-xL/Bax ratio, cytochrome c, cleaved caspase-9 and cleaved caspase-3 level, respectively. The protein/Actin ratio in control cells was set as 100%. All experiments were repeated at least 3 times. *P<0.05 vs control; ^#^ *P*<0.05 vs glutamate.

The results presented so far suggested that DAPK1-NMDA receptor interaction was critical but not the sole target of pyruvate. We hypothesized that Beclin-1 and DAPK1 interaction played a role in pyruvate mediated protection. Beclin-1 can form protein complex with both Bcl-xL and Bcl-2 proteins, which are key regulators of apoptosis [29]. Therefore, both of them were analyzed by western blot method. The results showed that Bcl-xL, rather than Bcl-2 was concentration dependently increased by pyruvate. Bcl-xL protects cells from cell death by inhibiting Bax-induced caspase activation via cytochrome c release from mitochondria [30]. Our results showed that Bax was significantly increased in glutamate treatment group. The increase in Bax was attenuated by pyruvate. The Bcl-xL/Bax ratio was reduced to 22.3% by glutamate. Pyruvate concentration dependently reversed the suppression of the ratio. When cells were challenged with glutamate, cytochrome c was raised to 166.5% compared with control cells. If cells were preincubated with pyruvate, the increased cytochrome c induced by glutamate was reversed. After treatment with pyruvate followed by glutamate insult, cleaved forms of caspase-9 and caspase-3 were detected. Activation of the caspase cascade was observed in glutamate group. Significant increases in cleaved caspase-9 and caspase-3 were observed after glutamate treatment. The extent of increase in cleaved caspase-9 was less than cleaved caspase-3. Pyruvate significantly reduced the cleaved caspase-3 and caspase-9 in all concentration groups (Fig. 4).

### Autophagy was Involved in the Protection of Pyruvate against Excitotoxicity

**Figure 5:**
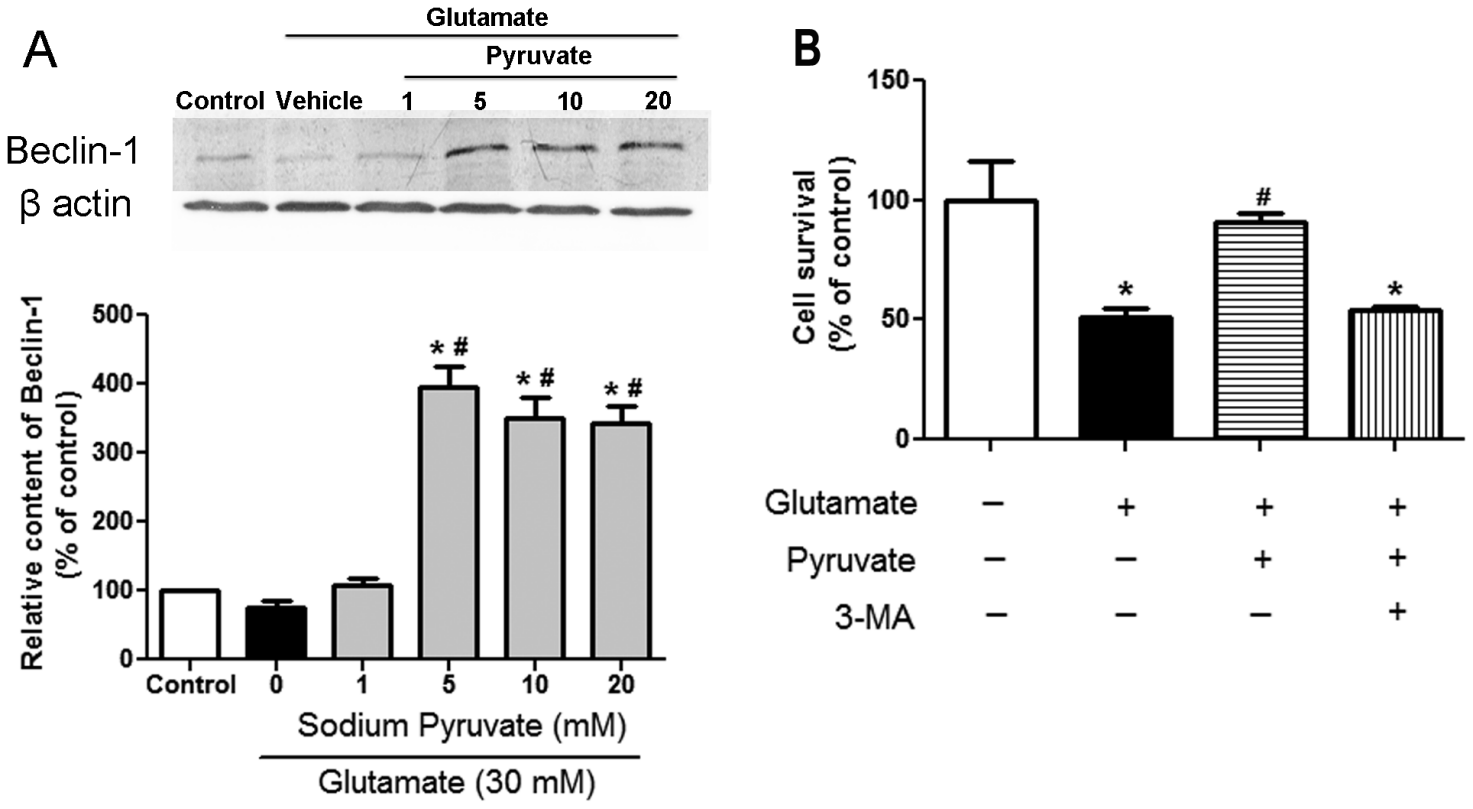
Pyruvate activated autophagy to protect cells. A, Cells were preincubated with vehicle or pyruvate (from 1 to 20 mM) for 30 minutes at 37°C. After preincubation, cells were challenged with vehicle or 30 mM glutamate for 3 hours. The Beclin-1 level was determined by western blot analysis. β-Actin was used as an internal control. The Beclin-1/Actin ratio in control cells was set as 100%. B, Cell viability was determined by MTT assay. After preincubation with pyruvate with or without 0.1 mM 3-methyladenine for 30 minutes, cells were challenged with 30 mM glutamate. Cell growth of control cells was set as 100%. *P<0.05 vs control; ^#^ *P*<0.05 vs glutamate; n = 6.

Beclin-1 is a biomarker of autophagy [31]. As Fig. 5 shown, the level of Beclin-1 remained constant in the presence or absence of glutamate. An increase in Beclin-1 level was observed when SH-SY5Y cells were pre-incubated with 5, 10 and 20 mM pyruvate. The increased Beclin-1 suggested that autophagy was involved in the pyruvate protection. To explore the effect of autophagy in the pyruvate protection, autophagy inhibitor 3-methyladenine was used. Cell survival rate was decreased from 91.1% to 53.9% when cells were pretreated with a combination of 0.1 mM 3-methyladenine and pyruvate. This indicated that pyruvate protection was mediated by autophagy.

### Mitochondrial Damage was Involved in the Protection of Pyruvate against Excitotoxicity

**Figure 6:**
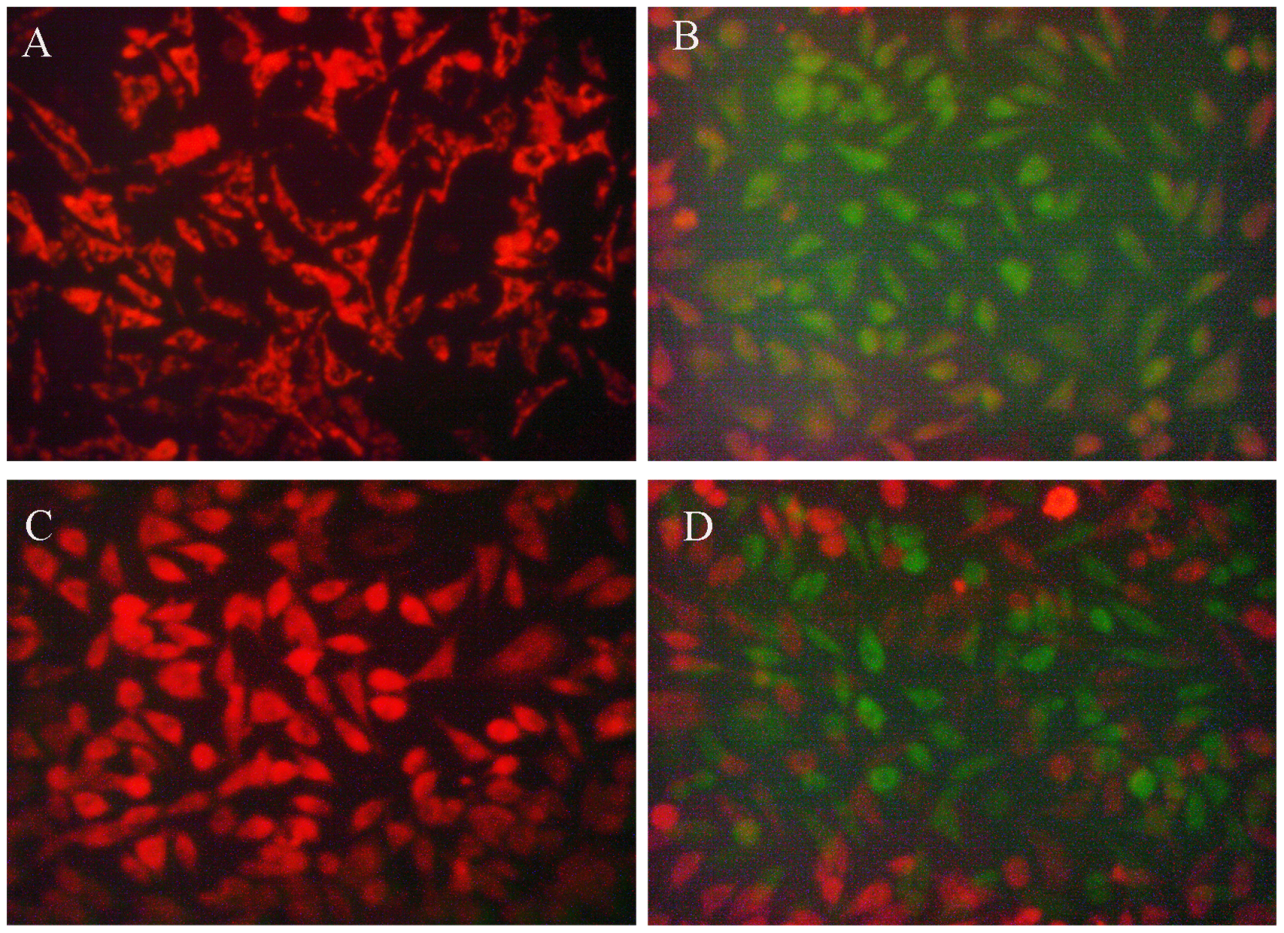
Mitochondrial membrane potential was determined. Mitochondrial membrane potential was determined the JC-1 miochondrial membrane potential assay kit. Red fluorescence represented high mitochondrial membrane potential while green fluorescence represented low mitochondrial membrane potential. A, Cells were treated with vehicle only. B, Cells were preincubated with vehicle followed by challenging with 30 mM glutamate for 3 hours. C, Cells were preincubated with 20 mM pyruvate for 30 minutes followed by challenging with 30 mM glutamate for 3 hours. D, Cells were pretreated with pyruvate and 0.1 mM 3-methyladenine for 3 hours followed by challenging with 30 mM glutamate for 3 hours. All cells were cultured at 37°C.

Cytochrome c release was implicated with mitochondrial damage [32]. Therefore, mitochondrial damage was determined by JC-1 which is a sensitive marker for mitochondrial membrane potential. JC-1 aggregates in the matrix and generates red fluorescence at high mitochondrial membrane potential (ΔΨm). Otherwise, JC-1 monomer generates green fluorescence at low ΔΨm. Thus, the increase in green and decrease in red fluorescence represent the loss of ΔΨm. As Fig. 6 shown, normal cells have a high ΔΨm identified by strong red fluorescence without green fluorescence. When cells were challenged with glutamate, almost all of them exhibited green fluorescence with little red fluorescence. In contrast to glutamate treatment group, cells in the pyruvate treatment group did not display stronger green fluorescence than control group, although the red fluorescence turned to weaker compared with control. The effects of pyruvate were partly reversed by co-incubation with 3-methyladenine. The results inferred that pyruvate prevented the dissipation of mitochondrial membrane potential induced by glutamate. This was partly mediated by autophagy.

## Discussion

Previous results have demonstrated that pyruvate has a protective role in CNS neurons through antioxidation [20], [21], anti-inflammation [22] and inducing EPO expression [17]. Our results underscore that pyruvate protect cells from glutamate excitotoxicity by disrupting the interaction between DAPK1 and NMDA receptors and enhancing the interaction between DAPK1 and Beclin-1.

DAPK1 is a calcium/calmodulin (CaM) serine/threonine (Ser/Thr) kinase which is a positive mediator of cell death [33]. In a rat stroke model, DAPK1 combines with NMDA receptors leading to NMDA receptors phosphorylation and over-activation [13]. Subsequently the intracellular calcium is overloaded which triggers the cell death [34]. Consistent with this finding, our results demonstrate that glutamate promotes DAPK1 and NMDA receptor interaction which results in NMDA receptor phosphorylation and over-activation. Furthermore, the protection of DAPK1 inhibitor indicates that DAPK1 is attributed to glutamate excitotoxicity. All these evidences suggest that DAPK1 and NMDA receptors interaction plays a very important role in the glutamate excitotoxicity. These results provide a rationale that disruption of DAPK1-NMDA receptor complex will be a method for glutamate excitotoxicity related disease. We have proved that pyruvate has potential to disrupt DAPK1 and NMDA receptors complex. To determine the role of DAPK1 in the pyruvate protective process, pharmacological intervene is used in the study instead of DAPK1 RNA interference (RNAi). As we know, RNAi is a post-transcriptional process which causes transcriptional repression [35]. DAPK1 is a crucial protein in stoke models [13]. When DAPK1 is specifically knocked down by RNAi, the excitotoxicity induced by NMDA is almost entirely blocked [36]. These circumstantial evidences suggest that under DAPK1 silenced conditions glutamate would not injure the cells any more. It therefore follows that pretreating DAPK1 silenced cells with pyruvate would confer a similar level from glutamate excitotoxicity as DAPK1 silence regardless of whether the protection by pyruvate is mediated by DAPK1. However, compared with DAPK1 RNAi, DAPK1 inhibitor can provide a moderate protection. If the protection of pyruvate does not involve DAPK1, the protective effect of pyruvate would not be affected by DAPK1 inhibitor. Our results show that in the presence of DAPK1 inhibitor, the protection of pyruvate is partly inhibited. These evidences indicate that DAPK1 complex regulation is mediated the pyruvate protective effects. The NMDA receptors related pathway further examined in the current study explore the specific protective mechanism of pyruvate. Pyruvate causes a decrease in NMDA receptors phosphorylation, which results in the blockade of calcium ion influx into intra-cellular stores. Calcium overload promotes the opening of the permeability transition pore. The increased permeability of the inner mitochondrial membrane leads to matrix swelling, rupture of the outer mitochondrial membrane, and finally results in the release of cytochrome c [37]. Our results indicate that Ca^2+^ independent cytochrome c release is also inhibited by pyruvate. In lethal conditions, Bax/Bak find their way to insert the outer mitochondria membrane [38]. This forms supramolecular openings resulting from the formation of mitochondrial permeability transition pores or from discontinuities in the membrane. The supramolecular opening mediates cytochrome c release [39]. Bcl-2 or Bcl-xL regulates Bax/Bak dependent death by inhibiting the release of cytochrome c from the mitochondrial inter-membrane space into the cytosol [30], [40], [41].The decreased Bcl-2 or Bcl-xL to Bax ratio causes Ca^2+^ independent cytochrome c release from mitochondria which is a trigger of apoptosis [38], [39]. The present results indicate that Bcl-xL, not Bcl-2, is involved in the protective process of pyruvate. Released cytochrome c from the mitochondria binds to Apaf-1 and procaspase-9 to lead to caspase-9 activation in the presence of dATP [42]. Activated caspase-9 in turn cleaves and activates caspase-3, causing cell death [43]. Inhibition of caspase-9 activation caused by pyruvate is not significant concentration dependent while inhibition of caspase-3 is concentration dependent. The seemingly contradiction should be attributed to caspase cascade amplification [44]. Of cause, caspase-3 activated through other pathway was not excluded. However, the results suggest that inhibition of caspase-9/caspase-3 activation at least in part mediates the pyruvate protection.

Beclin-1 has a central role in autophagy, which is a catabolic pathway by which protein and organelle undergo degradation [45], [46]. The autophagic process compasses six steps of initiation, nucleation, elongation, closure, maturation and degradation [47]. Beclin-1 associates with class III PI3K/p150 and drives nucleation [48]. Beclin-1 is cleaved by activated caspase-3 with two cleavage sites at positions 124 and 149 [49], [50]. Our result indicates that Beclin-1 is increased by pyruvate. The inhibition of caspase-3 activation at least in part attributes to the increase in Beclin-1 induced by pyruvate. Beclin-1 possesses a BH3 domain that interacts with the BH3 binding groove of both Bcl-2 and Bcl-xL [51]. Beclin-1 dependent autophagy is abolished after combination with Bcl-2/Bcl-xL [31]. Interestingly, our results indicate that the biomarker of autophagy Beclin-1 [31] and autophagy inhibitor Bcl-xL are simultaneously increased by pyruvate. Therefore, we speculate autophagy is involved in the protective effects of pyruvate. The autophagy inhibitor 3-methyladenin is used to verify whether autophagy plays a role in the protection of pyruvate. When 3-methyladenin is present, pyruvate does not protect cells against glutamate insult any more. These indicate that autophagy is involved in the pyruvate protection process. To our surprise, autophagy inhibitor 3-methyladenine significantly blocks the prevention of mitochondrial trans-membrane potential loss. This reveals that autophagy induced by pyruvate protects cells also through mitochondrial pathway. However, how the autophagy works in the mitochondrial protection of pyruvate requires more studies in future. The autophagy inhibitor Bcl-xL is increased while autophagy is activated by pyruvate at the same time. How could this happen? We note that pyruvate has a more potential protective effect for pyruvate against glutamate excitotoxicity than that of DAPK1 inhibitor. This suggests that other protective mechanism should exist except regulating DAPK1-NMDA receptor complex by pyruvate. As we known, DKPA 1 not only combines with NMDA receptors, but also interacts with Beclin-1 [14]. After combination with DAPK1, Beclin-1 is phosphorylated on its BH3 domain, which weakens its interactions with Bcl-XL [14], [52]. DAPK1 and Beclin-1 interaction causes Bcl-xL and Beclin-1 complex disassociation [53]. The present results indicate that regulation of DAPK1-Beclin-1 complex is involved in the process of pyruvate protection.

**Figure 7:**
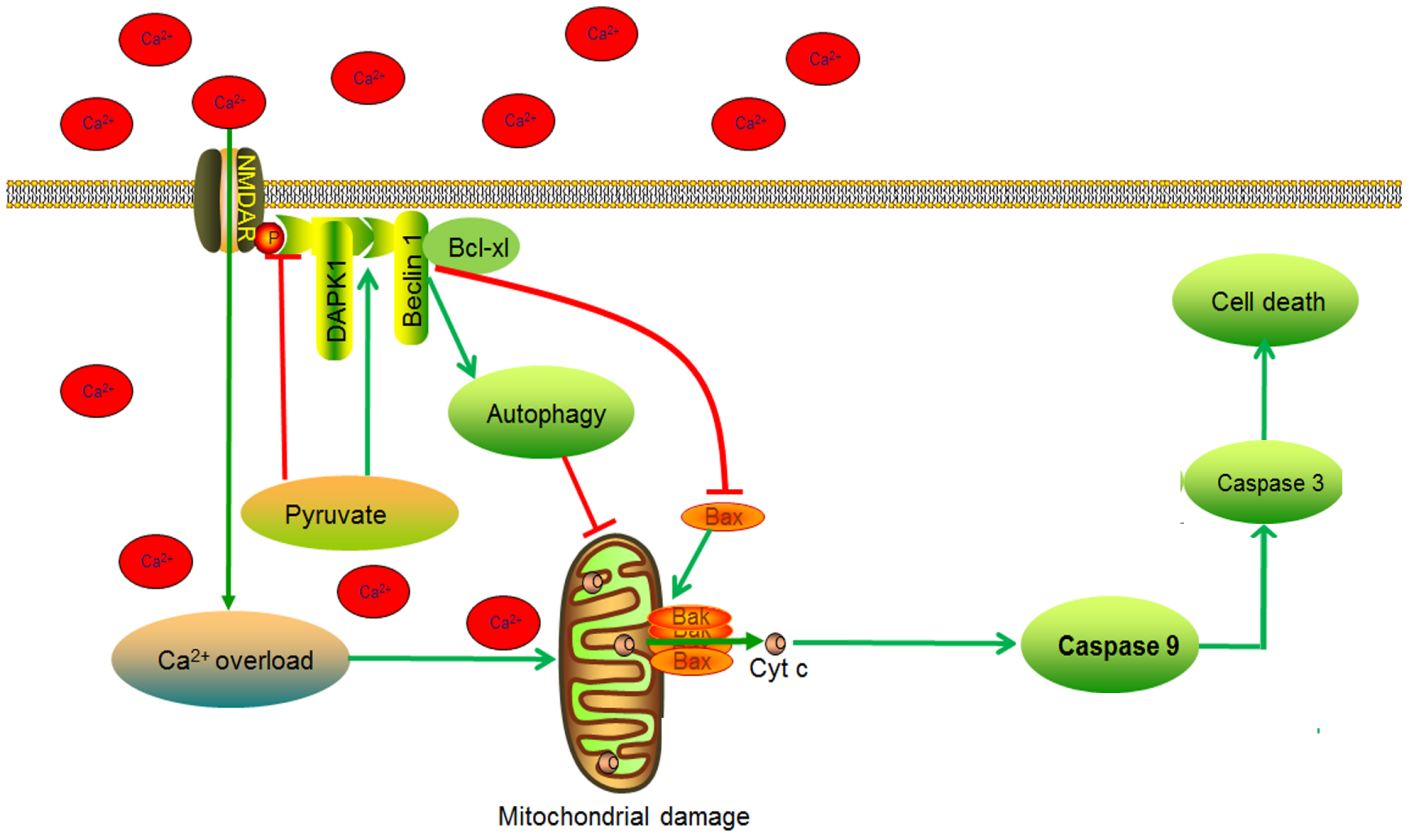
Overview of pyruvate protective mechanism. Pyruvate protects cells from glutamate excitotoxicity by regulating DAPK1 complexes through DAPK1 and NMDA receptors complex dissociation and DAPK1 and Beclin-1 association. They go forward to protect cells by attenuating Ca2+ overload and activating autophagy, respectively. Finally, a convergence of the two ways protects mitochondria from glutamate excitotoxicity, which leads to cell survival.

Taken together, pyruvate protects cells from glutamate excitotoxicity by regulating DAPK1 complexes through DAPK1 and NMDA receptors complex dissociation and DAPK1 and Beclin-1 association. They go forward to protect cells by attenuating Ca^2+^ overload and activating autophagy. Finally, the two ways converge to protect mitochondria from glutamate excitotoxicity, which leads to cell survival (Fig. 7). It should be noted that there are some limits in pyruvate development. A lot of studies indicates that the effective concentration of pyruvate is similar to us [17], [19], [20], which is too high to use in clinic. The poor stability in solution [54] leads to pyruvate breakdown. Therefore, high exposure of pyruvate is required to obtain the efficacy. The pretreatment is another deficiency in our study. We ever checked the role of pyruvate in reversing rather that preventing excitotoxicity. Unfortunately, the results were negative. Pyruvate could not rescue the cells from excitotoxicity (data not shown). After all pretreatment has its practical values which at least indicates the pound has a potential for disease prevention or preventing aggravation by protecting undamaged cells. We find that pyruvate exerts a strong neuroprotective effect against the glutamate excitotoxicity by regulating DAPK1 complex, which should be taken into account in drug discovery [16]. Though the pharmacological application of pyruvate is limited by its stability in solution [55], high working concentration and administration time, pyruvate has a potential to become a lead compound for regulating the new target DAPK1 complex to treat neurodegenerative disease which are caused by glutamate excitotoxicity. However, more work is clearly needed to develop pyruvate.
